# Supplementary material for: A structure-based tool to interpret the significance of kinase mutations in clinical next generation sequencing in cancer
Source: Front Oncol. 2025 Aug 4;15:1599389. doi: 10.3389/fonc.2025.1599389 (PMC12358288; doi:10.3389/fonc.2025.1599389)
Supplement: Supplementary file 1 [file DataSheet1.pdf]

A structure-based tool to interpret the significance of kinase mutations in clinical next generation sequencing in cancer

Amith Rangarajan[1], Ilona Sviezhentseva, PhD[2], Emma Gunderson[3], Yana Pikman, MD[2,4], Matthew P. Jacobson, PhD[1], Beth Apsel Winger, MD, PhD[3,5]

[1]Department of Pharmaceutical Chemistry, University of California San Francisco, San Francisco CA
[2]Department of Pediatric Oncology, Dana-Farber Cancer Institute, Boston, MA
[3]Department of Clinical Pharmacy, University of California San Francisco, San Francisco CA
[4]Division of Hematology/Oncology, Boston Children's Hospital, Boston, MA
[5]Department of Pediatrics, Division of Hematology, University of California San Francisco, San Francisco CA

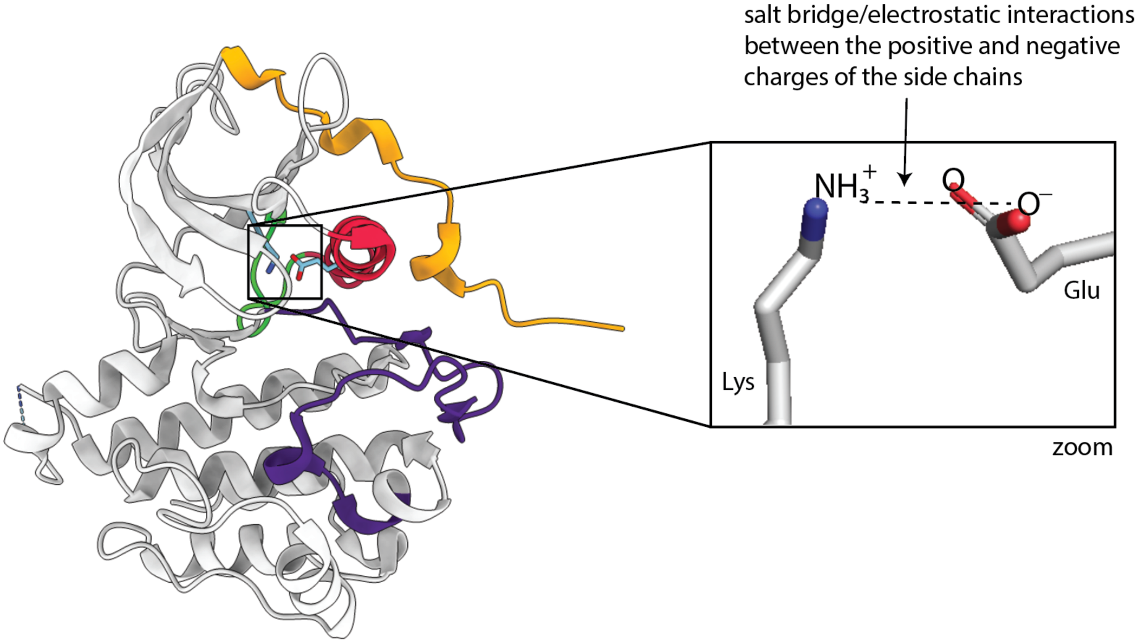

Figure S1. Electrostatic interactions between the αC-helix and the kinase core. This figure shows the salt bridge that is formed between the Glu on the αC-helix and the Lys in the core of the kinase. This salt bridge is essential for the catalytic activity of the kinase and enables phosphoryl transfer.

Table S1: Representative structures and the corresponding COSMIC data

Gene_name	Representative structure	Uniprot_id	RTK_Family	COSMIC_ID
ALK	2XP2	Q9UM73	ALK	COSG350
AXL	homology model	P30530	AXL	COSG10854
CSF1R	3KRJ	P07333	PDGFR	COSG107
DDR1	6FTO	Q08345	DDR	COSG9377
DDR2	homology model	Q16832	DDR	COSG139
EGFR	1M14	P00533	EGFR	COSG150
EPHA1	homology model	P21709	EPH	COSG152
EPHA10	homology model	Q5JZY3	EPH	COSG37048
EPHA2	5EK7	P29371	EPH	COSG155
EPHA3	2QOQ	P29320	EPH	COSG154
EPHA4	homology model	P54764	EPH	COSG153
EPHA5	2r2p	P54756	EPH	COSG22150
EPHA7	2REI	Q15375	EPH	COSG158
EPHA8	homology model	P29322	EPH	COSG159
EPHB1	5MIA	P54762	EPH	COSG160
EPHB2	3ZF7	P29323	EPH	COSG40191
EPHB3	5L6P	P54753	EPH	COSG162
EPHB4	6FNK	P54760	EPH	COSG163
EPHB6	homology model	O15197	EPH	COSG19012
ERBB2	3PP0	P04626	EGFR	COSG165
ERBB3	homology model	P21860	EGFR	COSG166
ERBB4	3BCE	Q15303	EGFR	COSG167
FGFR1	3AM6	P11362	FGFR	COSG171
FGFR2	3B2T	P21802	FGFR	COSG173
FGFR3	6PNX	P22607	FGFR	COSG97353
FGFR4	4QF5	P22455	FGFR	COSG1767
FLT1	3HNG	P17948	VEGFR	COSG187

FLT3	1T3	P36888	PDGFR	COSG210
IGF1R	5FXQ	P08069	InsR	COSG215
INSR	1IRK	P06213	InsR	COSG220
IRR	homology model	P14616	InsR	COSG219
KDR	4ASD	P35968	VEGFR	COSG615273
KIT	5GOE	P10721	PDGFR	COSG211
LTK	homology model	P29376	ALK	COSG91303
MERTK	7AB2	Q12866	Axl	COSG106715
MET	2G15	P08581	Met	COSG12
MST1R	3PLS	Q04917	Met	COSG315
NTRK1	6PL4	P04629	Trk	COSG336
NTRK2	4AT5	Q16620	Trk	COSG27120
NTRK3	4YMJ	Q16288	Trk	COSG9247
PDGFRA	5K5X	P16234	PDGFR	COSG13
RET	6NEC	P07949	Ret	COSG17
ROR1	6T9V	Q01973	Ror	COSG16
ROR2	3ZZW	Q01974	Ror	COSG417
RYK	6TUA	P34925	Ryk	COSG620600
TEK	1FVR	Q02763	Tie	COSG488
TIE1	homology model	P35590	Tie	COSG494
TYRO3	homology model	Q06418	Axl	COSG515

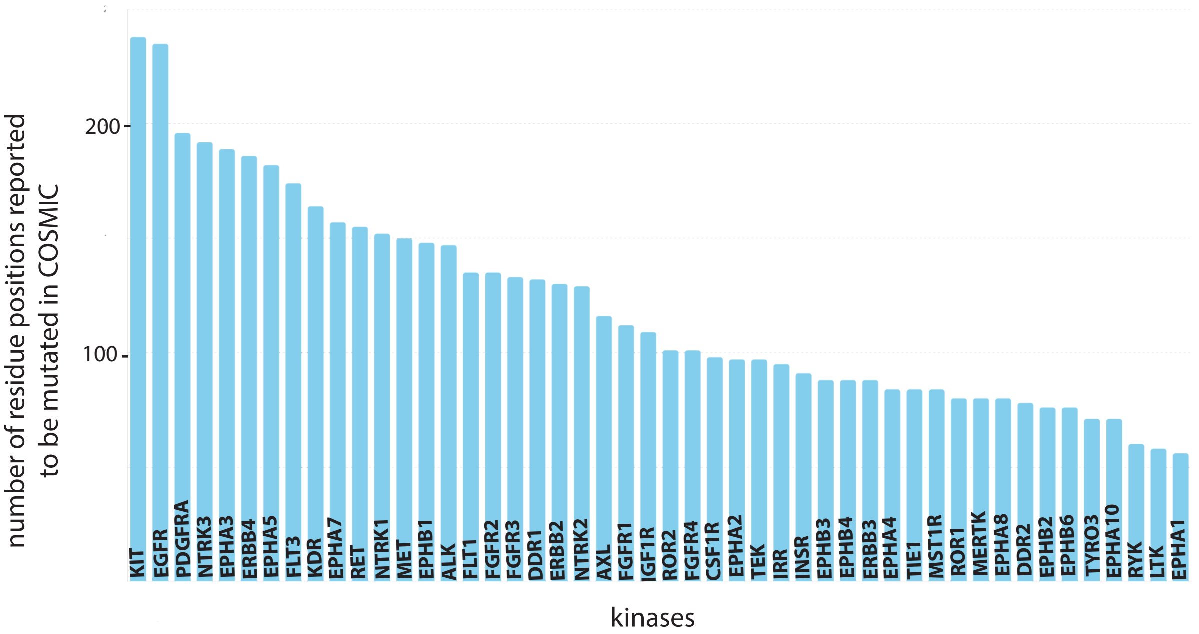

Figure S2. The distribution of residue positions with mutations across receptor tyrosine kinases. The dataset has a total of 5778 residue positions from 48 receptor tyrosine kinases.

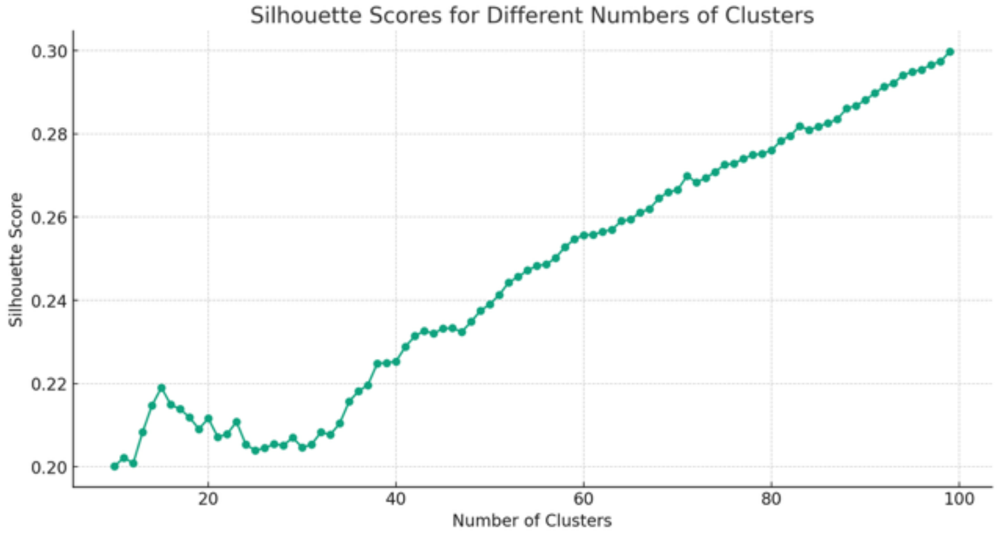

Figure S3. The silhouette score supported clustering the data into 40 clusters. The silhouette score is a measure between -1 and 1 that indicates how well each point fits within its assigned cluster. A score close to 1 means the point is well-matched to its cluster and far from others (ideal). Scores near 0 suggest overlap between clusters, and negative scores suggest misclassification. In general, a higher silhouette score means better clustering. The variation in the silhouette score using different numbers of clusters was minimal suggesting that there is no clearly optimal number of clusters. Increasing beyond 40 clusters modestly increased the score, but at the cost of greatly increased complexity of analysis and interpretation. Empirically, using other numbers of clusters, between 20-40, did not qualitatively change the interpretation.

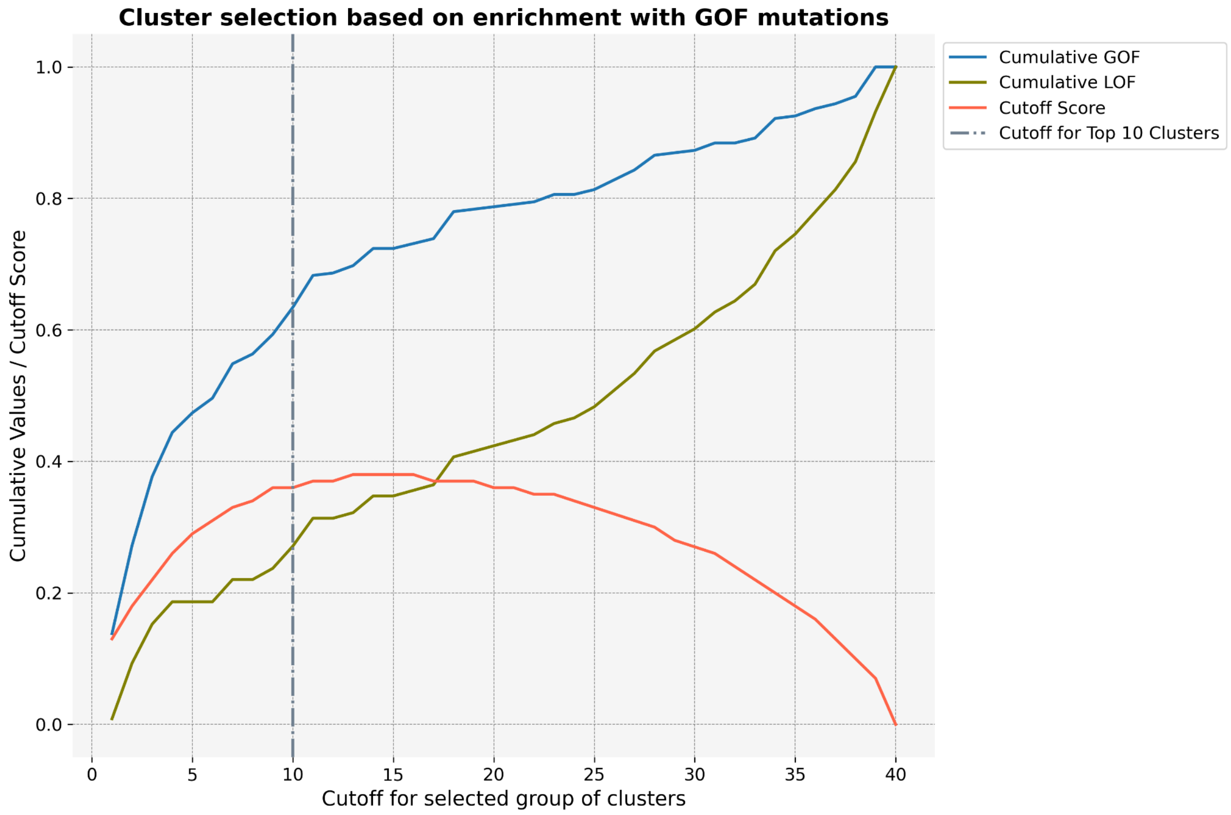

Figure S4. Ten clusters that maximized GOF mutations and had few LOF mutations were selected as the top ten clusters.

Supplemental Methods

Clustering

Hierarchical clustering was conducted using the Euclidean distance and Ward linkage method, with the X, Y, and Z coordinates of the side chain center of masses as the input parameters. We chose 40 clusters empirically. When generating fewer clusters, e.g., 20, the clusters encompass multiple key functional regions such as the activation loop and αC-helix. Using numbers of clusters larger than 40 generated very small clusters with only a few mutations, making it difficult to perform further analyses.

Next, a subset of clusters that were enriched in gain-of-function (GOF) mutations and minimized loss-of-function (LOF) mutations, were selected. The 'net_GOF' and 'net LOF' mutations for each cluster were calculated based on OncoKB annotations as the sum of GOF and likely GOF annotations, or LOF and likely LOF values, respectively as shown in eq 1,2.

$$\text{net GOF}_{(i)} = \text{GOF}_{(i)} + \text{likely GOF}_{(i)} \quad (1)$$

$$\text{net LOF}_{(i)} = \text{LOF}_{(i)} + \text{likely LOF}_{(i)} \quad (2)$$

These values were normalized to the total GOF or LOF (‘norm GOF’ and ‘norm LOF’), as follows:

$$\text{norm GOF}_{(\text{i})} = \frac{\text{net GOF}_{(\text{i})}}{\text{total GOF}} \quad (3)$$

$$\text{norm LOF}_{(\text{i})} = \frac{\text{net LOF}_{(\text{i})}}{\text{total LOF}} \quad (4)$$

where,

$$\text{total GOF} = \int_{\text{i}=1}^{40} \text{net GOF}_{(\text{i})} \quad (5)$$

$$\text{total LOF} = \int_{\text{i}=1}^{40} \text{net LOF}_{(\text{i})} \quad (6)$$

Cumulative values for GOF and LOF were calculated as shown in equations 7 and 8. A "sorting score" was calculated using the cumulative GOF and cumulative LOF as shown in equation 9.

$$\text{cumulative GOF}_{(\text{i})} = \int_{\text{j}=1}^{\text{i}} \text{norm GOF}_{(\text{j})} \quad \text{for i} = 1,2..40 \ (7)$$

$$\text{cumulative LOF}_{(\text{i})} = \int_{\text{j}=1}^{\text{i}} \text{norm LOF}_{(\text{j})} \quad \text{for i} = 1,2..40 \ (8)$$

$$\text{sorting score}_{(\text{i})} = \text{net GOF}_{(\text{i})} - \text{net LOF}_{(\text{i})} \ (9)$$

The clusters were then ranked based on those that contained the largest fraction of GOF relative to LOF mutations (Figure S3) as calculated by the cut-off score:

$$\text{cutoff score}_{(\text{i})} = \text{cumulative GOF}_{(\text{i})} - \text{cumulative LOF}_{(\text{i})} \quad (10)$$

which revealed that most GOF or likely GOF (LGOF) mutations, and few LOF or likely LOF (LLOF) mutations, were in the top 10 clusters.

Mutagenesis and plasmid preparation

The following mutagenesis primers were used:

Gene	Mutation	Forward Primer	Reverse Primer
PDGFRA	Y555C	AACAGAAACCGAGGTGTGAAATT CGCTGGAGGGTCAT	ATGACCCTCCAGCGAATTTCACA CCTCGGTTTCTGTT
	V561D	GAAATTCGCTGGAGGGACATTG AATCAATCAGCC	GGCTGATTGATTCAATGTCCCTC CAGCGAATTTC
	L580M	CTTGAGTCATAAGGCATCTGCAT CGGGTCCACA	TGTGGACCCGATGCAGATGCCT TATGACTCAAG
	Y849C	ACATCATGCATGATTCGA ACTGTGTGTCGA AAGGCAGTACCT TT	AAAGGTACTGCCTTTCGACACAC AGTTCGA ATCATGCATGATGT
	G652E	GAT AAT GAC TCA CCT GGA GCC ACA TTT GAA CAT TGT AA	TTA CAATGTTCA AATGTGGCTCCAGGTGAGTCATT A TC
FLT3	Y572C	TGTAGCTGGCTTTCACACCTAAA TTGCTTTTTGTACTTGTGACA	TGTCACAAGTACAAAAAGCAATT TAGGTGTGAAAGCCAGCTACA
	M578I	GCCGGTCACCTGTACTATCTGTA GCTGGCTT	AAGCCAGCTACAGATAGTACAG GTGACCGGC
	F594I	GAGATCATATTCATATTCTCTGAT ATCAACGTAGAAGTACTCATTAT CT	AGATAATGAGTACTTCTACGTTG ATATCAGAGAATATGAATATGAT CTC
	Y842C	GCCCCTGACAACACAGTTGGAA TCACTCATGATATCTCG	CGAGATATCATGAGTGATTCCAA CTGTGTTGTCAGGGGC
	G669E	CAATATTCTCGTGGCTTTCCAGC TGGGTCATCATC	GATGATGACCCAGCTGGAAAGC CACGAGAATATTG
	D835Y	GTTGGAATCACTCATGATATATC GAGCCAATCCAAAGTCAC	GTGACTTTGGATTGGCTCGATAT ATCATGAGTGATTCCAAC
