# Supplementary material for: A structure-based tool to interpret the significance of kinase mutations in clinical next generation sequencing in cancer
Source: Front Oncol. 2025 Aug 4;15:1599389. doi: 10.3389/fonc.2025.1599389 (PMC12358288; doi:10.3389/fonc.2025.1599389)
Supplement: Supplementary file 2 [file DataSheet2.pdf]

Appendix 1. Multiple sequence alignment. Secondary structure annotation based on EGFR.

```
                                                               η1    β1      TTT
EGFR
EGFR      1  ...............................GEAPNQALLRILKETEFKKIKVLGSGAFG
ALK       1  ........................NPNYCFAGKTSSISDLKEVPRKNITLIR..GLGAFG
AXL       1  .................................................LGKTLGEGEFG
CSF1R     1  .........PKYQVRWKIIESYEGNSYTFIDPTQLPYNEKWEFPRNNLQFGKTLGAGAFG
DDR1      1  ...................................DGPPRVDFPRSRLRFKEKLGEGQFG
DDR2      1  ...............................................LTFKEKLGEGQFG
EPHA1     1  .................................................VDTVIGEGEFG
EPHA10    1  ...................................................RSLGGGRFG
EPHA2     1  ....................KTYVDPHTYEDPNQAVLKFTTEIHPSCVTRQKVIGAGEFG
EPHA3     1  .......................TYVDPHEDPTQTVHEFAKELDATNISIDKVVGAGEFG
EPHA4     1  ...............................................IKIEKVIGVGEFG
EPHA5     1  ..................................AVHEFAKEIEASCITIERVIGAGEFG
EPHA7     1  .....................TYIDPETYEDPNRAVHQFAKELDASCIKIERVIGAGEFG
EPHA8     1  ...............................................IHIEKIIGSGDSG
EPHB1     1  .....................................EFAKEIDVSFVKIEEVIGAGEFG
EPHB2     1  .........................................EIDISCVKIEQVI....FG
EPHB3     1  ..............................................CVKIEEVIGAGEFG
EPHB4     1  ....................................REFAKEIDVSYVKIEEVIGAGEFG
EPHB6     1  ...............................................IKIEEVIGTGSFG
ERBB2     1  .................................APNQALLRILKETELRKVKVLGSGAFG
ERBB3     1  ...............................................LRKLKVLGSGVFG
ERBB4     1  ................................TAPNQAQLRILKETELKRVKVLGSGAFG
FGFR1     1  ..........................VAGVSEYELPEDPRWELPRDRLVLGKPLGE...G
FGFR2     1  ........................PMLAGVSEYELPEDPKWEFPRDKLTLGKPL....FG
FGFR3     1  ..............................SELELPADPKWELSRARLTLGKPLGEGAFG
FGFR4     1  ......................QDPLLAGLVSLDLPLDPLWEFPRDRLVLGKPLGEGCFG
FLT1      1  ........................EVPLDEQCERPYDASKWEFARERLKLGKSLGRGAFG
FLT3      1  ......QFRYESQLQMVQVTGSSDNEYFYVDFREYEYDLKWEFPRENLEFGKVLGSGAFG
IGF1R     1  ...........................SYFSAADVYVPDEWEVAREKITMSRELGQGSFG
INSR      1  ................................SSVFVPDEWEVSREKITLLRELGQGSFG
IRR       1  ...................................................RELGQGSFG
KDR       1  ....................DPDELPLDEHCERLPYDASKWEFPRDRLKLGKPLGRGAFG
KIT       1  GPTYKYLQKPMYEVQWKVVEEINGNNYVYIDPTQLPYDHKWEFPRNRLSFGKTLGAGAFG
LTK       1  ...............................................VTLLRALGHGAFG
MERTK     1  ............................SHMEELQNKLEDVVIDRNLLILGRILGEGEFG
MET       1  ...............LLQNTVHIDLSALNPELVQAVQHVVIGPSSLIVHFNEVIGRGHFG
MST1R     1  .........................RDLDSALLAEVKDVLIPHERVVTHSDRVIGKGHFG
NTRK1     1  ...............................GLQGHIACVHHIKRRDIVLKWELGEGAFG
NTRK2     1  .................................GAMDTFVQHIKRHNIVLKRELGEGAFG
NTRK3     1  ....................................GIHVQHIKRRDIVLKRELGEGAFG
PDGFRA    1  ........RYEIRWRVIESISPDGHEYIYVDPMQLPYDSRWEFPRDGLVLGRVLGSGAFG
RET       1  .......................GPLSLSVDAFKILEDPKWEFPRKNLVLGKTLGEGEFG
ROR1      1  ..................................KPKSKAKELPLSAVRFMEELGECAFG
ROR2      1  ........................................KEISLSAVRFMEELGEDRFG
RYK       1  ......................YPTLRSVTLLEAKGKVKDIAISRERITLKDVLQEGTFG
TEK       1  ....................................PTIYPVLDWNDIKFQDVIGEGNFG
TIE1      1  ...............................................ITFEDLIGEGNFG
TYRO3     1  ...............................................FTLGRMLGKGEFG
             1   5    10   15   20   25   30   35   40   45   50   55   60
```

```
                 β2                             β3               α1
EGFR
EGFR     30  TVYKGLWIPEGEKVK............IPVAIKELREAT.SPKANKEILDEAYVMASV.D
ALK      35  EVYEGQVSP..................LQVAVKTLPEVC.SEQDELDFLMEALIISKF.N
AXL      12  AVMEGQLNQ...DDSI...........LKVAVKTMKIAICTRSELEDFLSEAVCMKEF.D
CSF1R    52  KVVEATAFGLGKEDAV...........LKVAVKMLKSTA.HADEKEALMSELKIMSHLGQ
DDR1     26  EVHLCEVDSPQDLVSLDFPLNVRKGHPLLVAVKILRPDA.TKNARNDFLKEVKIMSRL.K
DDR2     14  EVHLCEVEGMEKFKDKDFALDVSANQPVLVAVKMLRADA.NKNARNDFLKEIKIMSRL.K
EPHA1    12  EVYRGTLRLPSQDC.............KTVAIKTLKDTS.PGGQWWNFLREATIMGQF.S
EPHA10   10  ELCCGCLQLPGRQE.............LLVAVHMLRDSA.SDSQRLGFLAEALTLGQF.D
EPHA2    41  EVYKGMLKTKE................VPVAIKTLKAGY.TEKQRVDFLGEAGIMGQF.S
EPHA3    38  EVCSGRLKLPSKKE.............ISVAIKTLKVGY.TEKQRRDFLGEASIMGQF.D
EPHA4    14  EVCSGRLKVPGKRE.............ICVAIKTLKAGY.TDKQRRDFLSEASIMGQF.D
EPHA5    27  EVCSGRLKLPGKRE.............LPVAIKTLKVGY.TEKQRRDFLGEASIMGQF.D
EPHA7    40  EVCSGRLKLPGKRD.............VAVAIKTLKVGY.TEKQRRDFLCEASIMGQF.D
EPHA8    14  EVCYGRLRVPGQRD.............VPVAIKALKAGY.TERQRRDFLSEASIMGQF.D
EPHB1    24  EVYKGRLKLPGKRE.............IXVAIKTLKAGY.SEKQRRDFLSEASIMGQF.D
EPHB2    16  EVCSGHLKLPGKRE.............IFVAIKTL.....TEKQRRDFLSEASIMGQF.D
EPHB3    15  EVCRGRLKQPGRRE.............VFVAIKTLKVGY.TERQRRDFLSEASIMGQF.D
EPHB4    25  EVCRGRLKAPGKKE.............SCVAIKTLKGGY.TERQRREFLSEASIMGQF.E
EPHB6    14  EVRQGRLQPRGRRE.............QTVAIQALWAGG.AESLQMTFLGRAAVLGQF.Q
ERBB2    28  TVYKGIWIPDGENVK............IPVAIKVLRENT.SPKANKEILDEAYVMAGV.G
ERBB3    14  TVHKGVWIPEGESIK............IPVCIKVIEDKS.GRQSFQAVTDHMLAIGSL.D
ERBB4    29  TVYKGIWVPEGETVK............IPVAIKILNET....KANVEFMDEALIMASM.D
FGFR1    32  QVVLAEAIGLDKDKPNRV.........TKVAVKMLKSDA.TEKDLSDLISEMEMMKMIGK
FGFR2    33  QVVMAEAVGIDKPKEA...........VTVAVKMLKDDA.TEKDLSDLVSEMEMMKMIGK
FGFR3    31  QVVMAEAIGIDKDRAAKP.........VTVAVKMLKDDA.TDKDLSDLVSEMEMMKMIGK
FGFR4    39  QVVRAEAFGMDQA..............STVAVKMLKDNA.SDKDLADLVSEMEVMKLIGR
FLT1     37  KVVQASAFGIKKSPTC...........RTVAVKMLKEGA.TASEYKALMTELKILTHIGH
FLT3     55  KVMNATAYGISKTGVS...........IQVAVKMLKEKA.DSSEREALMSELKMMTQLGS
IGF1R    34  MVYEGVAKGVVKDEPE...........TRVAIKTV.EAA.SMRERIEFLNEASVMKEF.N
INSR     29  MVYEGNARDIIKGEAE...........TRVAVKTVNESA.SLRERIEFLNEASVMKGF.T
IRR      10  MVYEGLARGLEAGEES...........TPVALKTVNELA.SPRECIEFLKEASVMKAF.K
KDR      41  QVIEADAFGIDKTATC...........RTVAVKMLKEGA.THSEHRALMSELKILIHIGH
KIT      61  KVVEATAYGLIKSDAA...........MTVAVKMLKPSA.HLTEREALMSELKVLSYLGN
LTK      14  EVYEGLVIGLPGDSSP...........LQVAIKTLPELC.SPQDELDFLMEALIISKF.R
MERTK    33  SVMEGNLKQEDGTS.............LKVAVKTM.....SQREIEEFLSEAACMKDF.S
MET      46  CVYHGTLLDNDGKK.............IHCAVKSLNRIT.DIGEVSQFLTEGIIMKDF.S
MST1R    36  VVYHGEYIDQAQNR.............IQCAIKSLSRIT.EMQQVEAFLREGLLMRGL.N
NTRK1    30  KVFLAECHNLLPEQDK...........MLVAVKALKEA..SESARQDFQREAELLTML.Q
NTRK2    28  KVFLAECYNLK................ILVAVKTLKDA..SDNARKDFHREAELLTNL.Q
NTRK3    25  KVFLAECYNLSPTKDK...........MLVAVKALKDP..TLAARKDFQREAELLTNL.Q
PDGFRA   53  KVVEGTAYGLSRSQPV...........MKVAVKMLKPTA.RSSEKQALMSELKIMTHLGP
RET      38  KVVKATAFHLKGRAGY...........TTVAVKMLKENA.SPSELRDLLSEFNVLKQV.N
ROR1     27  KIYKGHLYLPGMDHA............QLVAIKTLKDYN.NPQQWMEFQQEASLMAEL.H
ROR2     21  KVYKGHLFGPAPGEQT...........QAVAIKTLKDKA.EGPLREEFRHEAMLRARL.Q
RYK      39  RIFHGILIDEKQ.................AFVKTVKDQA.SEIQVTMMLTESCKLRGL.H
TEK      25  QVLKARIKKDGLR..............MDAAIKRM.......KEYRDFAGELEVLCKLGH
TIE1     14  QVIRAMIKKDGLK..............MNAAIKMLKEYA.SENDHRDFAGELEVLCKLGH
TYRO3    14  SVREAQLKQEDGSF.............VKVAVKMLKADIIASSDIEEFLREAACMKEF.D
                 65   70   75   80   85   90   95  100  105  110  115  120
```

```
                                 β4             β5              α2         η2
EGFR             TT                                        TT

EGFR      76  NPHVCRLLGICL..TST.....VQLITQLMPFGCLLDYVREHK........DN.......
ALK       75  HQNIVRCIGVSLQ.SLP.....RFILLELMAGGDLKSFLRETR........PR.......
AXL       57  HPNVMRLIGVCFQGSERESFPAPVVILPFMKHGDLHSFLLYSR........LG.......
CSF1R    100  HENIVNLLGACTH.GGP.....VLVITEYCCYGDLLNFLRRKR........PP.......
DDR1      84  DPNIIRLLGVCVQ.DDP.....LCMITDYMENGDLNQFLSAHQ........LE.......
DDR2      72  DPNIIHLLAVCIT.DDP.....LCMITEYMENGDLNQFLSRHE........PP.......
EPHA1     57  HPHILHLEGVVTK.RKP.....IMIITEFMENGALDAFLRERE........DQ.......
EPHA10    55  HSHIVRLEGVVTR.GST.....LMIVTEYMSHGALDGFLRRHE........GQ.......
EPHA2     83  HHNIIRLEGVISK.YKP.....MMIITEYMENGALDKFLREKD........GE.......
EPHA3     83  HPNIIRLEGVVTK.SKP.....VMIVTEYMENGSLDSFLRKHD........AQ.......
EPHA4     59  HPNIIHLEGVVTK.CKP.....VMIITEYMENGSLDAFLRKND........GR.......
EPHA5     72  HPNIIHLEGVVTK.SKP.....VMIVTEYMENGSLDTFLKKND........GQ.......
EPHA7     85  HPNVVHLEGVVTR.GKP.....VMIVIEFMENGALDAFLRKHD........GQ.......
EPHA8     59  HPNIIRLEGVVTR.GRL.....AMIVTEYMENGSLDTFLRTHD........GQ.......
EPHB1     69  HRNIIRLEGVVTK.SRP.....VMIITEFMENGALDSFLRQND........GQ.......
EPHB2     57  HPNVIHLEGVVTK.STP.....VMIITEFMENGSLDSFLRQND........GQ.......
EPHB3     60  HPNIIRLEGVVTK.SRP.....VMILTEFMENCALDSFLRLND........GQ.......
EPHB4     70  HPNIIRLEGVVTN.SMP.....VMILTEFMENGALDSFLRLND........GQ.......
EPHB6     59  HPNILRLEGVVTK.SRP.....LMVLTEFMELGPLDSFLRQRE........GQ.......
ERBB2     74  SPYVSRLLGICL..TST.....VQLVTQLMPYGCLLDHVRENR........GR.......
ERBB3     60  HAHIVRLLGLCP..GSS.....LQLVTQYLPLGSLLDHVRQHR........GA.......
ERBB4     72  HPHLVRLLGVCL..SPT.....IQLVTQLMPHGCLLEYVHEHK........DN.......
FGFR1     82  HKNIINLLGACTQ.DGP.....LYVIVEYASKGNLREYLQARR........PP.......
FGFR2     81  HKNIINLLGACTQ.DGP.....LYVIVEYASKGNLREYLRARR........PP.......
FGFR3     81  HKNIINLLGACTQ.GGP.....LYVLVEYAAKGNLREFLRARR........PP.......
FGFR4     84  HKNIINLLGVCTQ.EGP.....LYVILECAAKGNLREFLRARR........PP.......
FLT1      85  HLNVVNLLGACTKQGGP.....LMVIVEYCKYGNLSNYLKSKR........KE.......
FLT3     103  HENIVNLLGACTL.SGP.....IYLIFEYCCYGDLLNYLRSKR........EK.......
IGF1R     80  CHHVVRLLGVVSQ.GQP.....TLVIMELMTRGDLKSYLRSLR........PE.......
INSR      76  CHHVVRLLGVVSK.GQP.....TLVVMELMAHGDLKSYLRSLR........PE.......
IRR       57  CHHVVRLLGVVSQ.GQP.....TLVIMELMTRGDLKSHLRSLR........PE.......
KDR       89  HLNVVNLLGACTKPGGP.....LMVIVEFCKFGNLSTYLRSKR........NE.......
KIT      109  HMNIVNLLGACTI.GGP.....TLVITEYCCYGDLLNFLRRKRDSFICSKTSP.......
LTK       61  HQNIVRCVGLSLR.ATP.....RLILLELMSGGDMKSFLRHSR........PH.......
MERTK     74  HPNVIRLLGVCIE.MSSQGIPKPMVILPFMKYGDLHTYLLYSR........LE.......
MET       91  HPNVLSLLGICLRSEGS.....PLVVLPYMKHGDLRNFIRNET........HN.......
MST1R     81  HPNVLALIGIMLPPEGL.....PHVLLPYMCHGDLLQFIRSPQ........RN.......
NTRK1     76  HQHIVRFFGVCTE.GRP.....LLMVFEYMRHGDLNRFLRSHG........PD.......
NTRK2     69  HEHIVKFYGVCVE.GDP.....LIMVFEYMKHGDLNKFLRAHG........PD.......
NTRK3     71  HEHIVKFYGVCG..DGP.....LIMVFEYMKHGDLNKFLRAHG........PG.......
PDGFRA   101  HLNIVNLLGACTK.SGP.....IYIITEYCFYGDLVNYLHKNR........DSFLSHSML
RET       85  HPHVIKLYGACSQ.DGP.....LLLIVEYAKYGSLRGFLRESR........KV.......
ROR1      73  HPNIVCLLGAVTQ.EQP.....VCMLFEYINQGDLHEFLIMRS.................
ROR2      68  HPNVVCLLGVVTK.DQP.....LSMIFSYCSHGDLHEFLVMRS........PD.......
RYK       80  HRNLLPITHVCIEEGEK.....PMVILPYMNWGNLKLFLRQCK........LV.......
TEK       64  HPNIINLLGACEH.RGY.....LYLAIEYAPHGNLLDFLRKSR...VLETDPA.......
TIE1      59  HPNIINLLGACKN.RGY.....LYIAIEYAPYGNLLDFLRKSR........VL.......
TYRO3     60  HPHVAKLVGVSLRSRAKGRLPIPMVILPFMKHGDLHAFLLASR.......IGE.......
                 125  130   135   140   145   150   155   160   165   170   175   180

                                          α3                β6      η3   β7
EGFR                                                                          TT

EGFR     114  ..............IGSQYLLNWCVQIAKGMNYLEDRRLVHRDLAARNVLV...KTPQH
ALK      114  ...........PSQSLAMLDLLHVARDIACGCQYLEENHFIHRDIAARNCLLTCPGPGRV
AXL      102  ..........DQPVYLPTQMLVKFMADIASGMEYLSTKRFIHRDLAARNCML...NENMS
CSF1R    139  ............GQLSSRDLLHFSSQVAQGMAFLASKNCIHRDVAARNVLL...TNGHV
DDR1     123  .....DKAAEGADPTISYPMLLHVAAQIASGMRYLATLNFVHRDLATRNCLV...GENFT
DDR2     111  ......NSSSSDVRTVSYTNLKFMATQIASGMKYLSSLNFVHRDLATRNCLV...GKNYT
EPHA1     96  ..............LVPGQLVAMLQGIASGMNYLSNHNYVHRDLAARNILV...NQNLC
EPHA10    94  ..............LVAGQLMGLLPGLASAMKYLSEMGYVHRGLAARHVLV...SSDLV
EPHA2    122  ..............FSVLQLVGMLRGIAAGMKYLANMNYVHRDLAARNILV...NSNLV
EPHA3    122  ..............FTVIQLVGMLRGIASGMKYLSDMGYVHRDLAARNILI...NSNLV
EPHA4     98  ..............FTVIQLVGMLRGIGSGMKYLSDMSYVHRDLAARNILV...NSNLV
EPHA5    111  ..............FTVIQLVGMLRGISAGMKYLSDMGYVHRDLAARNILI...NSNLV
EPHA7    124  ..............FTVIQLVGMLRGIAAGMRYLADMGYVHRDLAARNILV...NSNLV
EPHA8     98  ..............FTIMQLVGMLRGVGAGMRYLSDLGYVHRDLAARNVLV...DSNLV
EPHB1    108  ..............FTVIQLVGMLRGIAAGMKYLSEMNYVHRDLAARNILV...NSNLV
EPHB2     96  ..............FTVIQLVGMLRGIAAGMKYLADMNYVHRDLAARNILV...NSNLV
EPHB3     99  ..............FTVIQLVGMLRGIAAGMKYLSEMNYVHRDLAARNILV...NSNLV
EPHB4    109  ..............FTVIQLVGMLRGIASGMRYLAEMSYVHRDLAARNILV...NSNLV
EPHB6     98  ..............FSSLQLVAMQRGVAAAMQYLSSFAFVHRSLSAHSVLV...NSHLV
ERBB2    112  ..............LGSQDLLNWCMQIAKGMSYLEDVRLVHRDLAARNVLV...KSPNH
ERBB3     98  ..............LGPQLLLNWGVQIAKGMYYLEEHGMVHRNLAARNVLL...KSPSQ
ERBB4    110  ..............IGSQLLLNWCVQIAKGMMYLEERRLVHRDLAARNVLV...KSPNH
FGFR1    121  ...........GEEQLSSKDLVSCAYQVARGMEYLASKKCIHRDLAARNVLV...TEDNV
FGFR2    120  ..............QMTFKDLVSCTYQLARGMEYLASQKCIHRDLTARNVLV...TENNV
FGFR3    120  ..........GLDPEEQLTFKDLVSCAYQVARGMEYLASQKCIHRDLAARNVLV...TEDNV
FGFR4    123  ............GPLSFPVLVSCAYQVARGMQYLESRKCIHRDLAARNVLV...TEDNV
FLT1     125  ..............PITMEDLISYSFQVAKGMEFLSSRKCIHRDLAARNILL...SENNV
FLT3     142  ...........FSNVLTFEDLLCFAYQVAKGMEFLEFKSCVHRDLAARNVLV...THGKV
IGF1R    119  ........MNPVLAPPSLSKMIQMAGEIADGMAYLNANKFVHRDLAARNCMV...AEDFT
INSR     115  ......AENNPGRPPPTLQEMIQMAAEIADGMAYLNAKKFVHRDLAARNCMV...AHDFT
IRR       96  ......AENNPGLPQPALGEMIQMAGEIADGMAYLAANKFVHRDLAARNCMV...SQDFT
KDR      129  .......FVPYKDFLTLEHLICYSFQVAKGMEFLASRKCIHRDLAARNILL...SEKNV
KIT      156  ......AIMEDDELALDLEDLLSFSYQVAKGMAFLASKNCIHRDLAARNILL...THGRI
LTK      100  .........LGQPSPLVMRDLLQLAQDIAQGCHYLEENHFIHRDIAARNCLLSCAGPSRV
MERTK    118  ...........TGPRHIPLQTLLRFMVDIALGMEYLSNRNFLHRDLAARNCML...RDDMT
MET      131  ..............PTVKDLIGFGLQVAKGMKYLASKKFVHRDLAARNCML...DEKFT
MST1R    121  ..............PTVKDLISFGLQVARGMEYLAEQKFVHRDLAARNCML...DESFT
NTRK1    115  ....AAGGEDVAPGPLGLGQLLAVASQVAAGMVYLAGLHFVHRDLATRNCLV...GQGLV
NTRK2    108  ...AVLMAEGNPPTELTQSQMLHIAQQIAAGMVYLASQHFVHRDLATRNCLV...GENLL
NTRK3    109  ..............ELGLSQMLHIASQIASGMVYLASQHFVHRDLATRNCLV...GANLL
PDGFRA   147  DSEVKNLLSDDNSEGLTLLDLLSFTYQVARGMEFLASKNCVHRDLAARNVLL...AQGKI
RET      124  ............ERALTMGDLISFAWQISQGMQYLAEMKLVHRDLAARNILV...AEGRK
ROR1     110  ..............LDHGDFLHIAIQIAAGMEYLSSHFFVHKDLAARNILI...GEQLH
ROR2     107  ....................FVHLVAQIAAGMEYLSSHHVVHKDLATRNVLV...YDKLN
RYK      120  .......EANNPQAISQQDLVHMAIQIACGMSYLARREVIHKDLAARNCVI...DDTLQ
TEK      108  .....FAIANSTASTLSSQQLLHFAADVARGMDYLSQKQFIHRDLAARNILV...GENYV
TIE1      98  ETDPAFAREHGTASTLSSRQLLRFASDAANGMQYLSEKQFVHRDLAARNVLV...GENLA
TYRO3    106  ..........NPFNLPLQTLIRFMVDIACGMEYLSSRNFIHRDLAARNCML...AEDMT
                 185  190   195   200   205   210   215   220   225   230   235   240
```

```
                β8       TT   β9       β10              η4      α4      β11      α5
EGFR
EGFR     156  VKITDFGLAKLLGAEEKEYHAEGGKV...PIKWMALESILHRIYTHQSDVWSYGVTVWEL
ALK      163  AKIGDFGMARDIYRAML............PVKWMPPEAFMEGIFTSKTDTWSFGVLLWEI
AXL      149  VCVADFGLSKKIY................PVKWIAIESLADRVYTSKSDVWSFGVTMWEI
CSF1R    183  AKIGDFGLARDIMNDSNYIVKGARL....PVKWMAPESIFDCVYTVQSDVWSYGILLWEI
DDR1     175  IKIADFGMSRNLYAGDYYRVQGRAVL...PIRWMAWECILMGKFTTASDVWAFGVTLWEV
DDR2     162  IKIADFGMSRNLY................PIRWMSWESILLGKFTTASDVWAFGVTLWET
EPHA1    138  CKVSDFGLTRLL.................PIRWTAPEAIAHRIFTTASDVWSFGIVMWEV
EPHA10   136  CKISGFGRGPR..................PALWAAPETLQFGHFSSASDVWSFGIIMWEV
EPHA2    164  CKVSDFGLSRVLEDDPEATYTTSGGKI..PIRWTAPEAISYRKFTSASDVWSFGIVMWEV
EPHA3    164  CKVSDFG......................PIRWTSPEAIAYRKFTSASDVWSYGIVLWEV
EPHA4    140  CKVSDFGMSRVLE................PIRWTAPEAIAYRKFTSASDVWSFGIVMWEV
EPHA5    153  CKVSDFGLSRVLEDDPAAYTTRGKI....PIRWTAPEAIAFRKFTSASDVWSYGIVMWEV
EPHA7    166  CKVSDFGL.....................PVRWTAPEAIQYRKFTSASDVWSYGIVMWEV
EPHA8    140  CKVSDFGLSRVLE................PIRWTAPEAIAFRTFSSASDVWSFGVVMWEV
EPHB1    150  CKVSDFGLSRYLPTXTSS...........PVRWTAPEAIAYRKFTSASDVWSYGIVMWEV
EPHB2    138  CKVSDF.......................PIRWTAPEAIQYRKFTSASDVWSYGIVMWEV
EPHB3    141  CKVSDFGLSRFLEDDPSDPTYTSSLGGKIPIRWTAPEAIAYRKFTSASDVWSYGIVMWEV
EPHB4    151  CKVSDFGLSRFLEEGKI............PIRWTAPEAIAFRKFTSASDVWSYGIVMWEV
EPHB6    140  CKVARLGHS....................PLRWAAPEVIAHGKHTTSSDVWSFGILMWEV
ERBB2    154  VKITDFGLARLLDIDETEYHAGKV.....PIKWMALESILRRRFTHQSDVWSYGVTVWEL
ERBB3    140  VQVADFGVADLLP................PIKWMALESIHFGKYTHQSDVWSYGVTVWEL
ERBB4    152  VKITDFGLARLLEGDEKEYNAGKM.....PIKWMALECIHYRKFTHQSDVWSYGVTIWEL
FGFR1    167  MKIADFGLARDIHHIDYYKKTTNGRL...PVKWMAPEALFDRIYTHQSDVWSFGVLLWEI
FGFR2    163  MKIADFGLARDINNIDYYKKTTNGRL...PVKWMAPEALFDRVYTHQSDVWSFGVLMWEI
FGFR3    169  MKIADFGLARDVHNLDYYKKTTNGRL...PVKWMAPEALFDEVYTHQSDVWSFGVLLWEI
FGFR4    167  MKIADFGLAL...................PVKWMAPEALFDEVYTHQSDVWSFGILLWEI
FLT1     168  VKICDFGLARDIYKNPDYVRKTRL.....PLKWMAPESIFDKIYSTKSDVWSYGVLLWEI
FLT3     188  VKICDFGLARDIMSDSNYVVRGNARL...PVKWMAPESLFEGIYTIKSDVWSYGILLWEI
IGF1R    168  VKIGDFGMTRDIYETDYYR...KGLL...PVRWMSPESLKDGVFTTYSDVWSFGVVLWEI
INSR     166  VKIGDFGMTRDIYETDYYRKGGKGLL...PVRWMAPESLKDGVFTTSSDMWSFGVVLWEI
IRR      147  VKIGDFGMTRDVY................PVRWMAPESLKDGIFTTHSDVWSFGVVLWEI
KDR      179  VKICDFGLARDIYKDPDYVRKGDARL...PLKWMAPETIFDRVYTIQSDVWSFGVLLWEI
KIT      207  TKICDFGLARDIKNDSNYVVKGNARL...PVKWMAPESIFNCVYTFESDVWSYGIFLWEL
LTK      151  AKIGDFGMARDIY................PVKWMPPEAFLEGIFTSKTDSWSFGVLLWEI
MERTK    165  VCVADF.......................PVKWIAIESLADRVYTSKSDVWAFGVTMWEI
MET      173  VKVADFGLARDMYDKEYYSVHNKTGAKL.PVKWMALESLQTQKFTTKSDVWSFGVLLWEL
MST1R    163  VKVADFGLARDILDREYYSVQQHRHARL.PVKWTALESLQTYRFTTKSDVWSFGVLLWEL
NTRK1    168  VKIGDFGMSRDIYXTDYYRVGGRTML...PIRWMPPESILYRKFTTESDVWSFGVVLWEI
NTRK2    162  VKIGDFGMSRDVYSTDYYRVGGHTML...PIRWMPPESIMYRKFTTESDVWSLGVVLWEI
NTRK3    152  VKIGDFGMSRDVYXTDYYR...HTML...PIRWMPPESIMYRKFTTESDVWSFGVILWEI
PDGFRA   204  VKICDFGLARDIMHDSNYVSKGSTFL...PVKWMAPESIFDNLYTTLSDVWSYGILLWEI
RET      169  MKISDFGLSRDVSQGRI............PVKWMAIESLFDHIYTTQSDVWSFGVLLWEI
ROR1     152  VKISDLGLSREIYSADYYR....SLL...PIRWMPPEAIMYGKFSSDSDIWSFGVVLWEI
ROR2     144  VKISDLGLFREVYAADYYKLLGNSLL...PIRWMAPEAIMYGKFSIDSDIWSYGVVLWEV
RYK      169  VKITDNALSRDLFPMDYHCLENR......PVRWMALESLVNNEFSSASDVWAFGVTLWEL
TEK      160  AKIADFGLSRGQEVYVKKRL.........PVRWMAIESLNYSVYTTNSDVWSYGVLLWEI
TIE1     155  SKIADFGLSRGE.................PVRWMAIESLNYSVYTTKSDVWSFGVLLWEI
TYRO3    152  VCVADFGLSRKIY................PVKWLALESLADNLYTVQSDVWAFGVTMWEI
                 245  250  255  260  265  270  275  280  285  290  295  300
```

```
                             α6                                α7        η5
EGFR                 TT                                TT
EGFR     213  MTFG.SKPYDGIP.ASEISSILEKGER.......LPQPPICTIDVYMIMVKCWMIDADSR
ALK      211  FSLG.YMPYPSKS.NQEVLEFVTSGGR.......MDPPKNCPGPVYRIMTQCWQHQPEDR
AXL      193  ATRG.QTPYPGVE.NSEIYDYLRQGNR.......LKQPADCLDGLYALMSRCWELNPQDR
CSF1R    239  FSLG.LNPYPGILVNSKFYKLVKDGYQ.......MAQPAFAPKNIYSIMQACWALEPTHR
DDR1     232  LMLCRAQPFGQLT.DEQVIENAGEFFRDQGRQVYLSRPPACPQGLYELMLRCWSRESEQR
DDR2     206  FTFCQEQPYSQLS.DEQVIENTGEFFRDQGRQTYLPQPAICPDSVYKLMLSCWRRDTKNR
EPHA1    181  LSFG.DKPYGEMS.NQEVMKSIEDGYR.......LPPPVDCPAPLYELMKNCWAYDRARR
EPHA10   178  MAFG.ERPYWDMS.GQDVIKAVEDGFR.......LPPPRNCPNLLHRLMLDCWQKDPGER
EPHA2    222  MTYG.ERPYWELS.NHEVMKAINDGFR.......LPTPMDCPSAIYQLMMQCWQQERARR
EPHA3    202  MSYG.ERPYWEMS.NQDVIKAVDEGYR.......LPPPMDCPAALYQLMLDCWQKDRNNR
EPHA4    184  MSYG.ERPYWDMS.NQDVIKAIEEGYR.......LPPPMDCPIALHQLMLDCWQKERSDR
EPHA5    209  VSYG.ERPYWEMT.NQDVIKAVEEGYR.......LPSPMDCPAALYQLMLDCWQKERNSR
EPHA7    205  MSYG.ERPYWDMS.NQDVIKAIEEGYR.......LPAPMDCPAGLHQLMLDCWQKERAER
EPHA8    184  LAYG.ERPYWNMT.NRDVISSVEEGYR.......LPAPMGCPHALHQLMLDCWHKDRAQR
EPHB1    199  MSFG.ERPYWDMS.NQDVINAIEQDYR.......LPPPMDCPAALHQLMLDCWQKDRNSR
EPHB2    175  MSYG.ERPYWDMT.NQDVINAIEQDYR.......LPPPMDCPSALHQLMLDCWQKDRNHR
EPHB3    201  MSYG.ERPYWDMS.NQDVINAVEQDYR.......LPPPMDCPTALHQLMLDCWVRDRNLR
EPHB4    199  MSFG.ERPYWDMS.NQDVINAIEQDYR.......LPPPPDCPTSLHQLMLDCWQKDRNAR
EPHB6    180  MSYG.ERPYWDMS.EQEVLNAIEQEFR.......LPPPPGCPPGLHLLMLDTWQKDRARR
ERBB2    209  MTFG.AKPYDGIP.AREIPDLLEKGER.......LPQPPICTIDVYMIMVKCWMIDSECR
ERBB3    184  MTFG.AEPYAGLR.LAEVPDLLEKGER.......LAQPQICTIDVYMVMVKCWMIDENIR
ERBB4    207  MTFG.GKPYDGIP.TREIPDLLEKGER.......LPQPPICTIDVYMVMVKCWMIDADSR
FGFR1    224  FTLG.GSPYPGVP.VEELFKLLKEGHR.......MDKPSNCTNELYMMMRDCWHAVPSQR
FGFR2    220  FTLG.GSPYPGIP.VEELFKLLKEGHR.......MDKPANCTNELYMMMRDCWHAVPSQR
FGFR3    226  FTLG.GSPYPGIP.VEELFKLLKEGHR.......MDKPANCTHDLYMIMRECWHAAPSQR
FGFR4    208  FTLG.GSPYPGIP.VEELFSLLREGHR.......MDRPPHCPPELYGLMRECWHAAPSQR
FLT1     223  FSLG.GSPYPGVQMDEDFCSRLREGMR.......MRAPEYSTPEIYQIMLDCWHRDPKER
FLT3     245  FSLG.VNPYPGIPVDANFYKLIQNGFK.......MDQPFYATEEIYIIMQSCWAFDSRKR
IGF1R    222  ATLA.EQPYQGLS.NEQVLRFVMEGGL.......LDKPDNCPDMLFELMRMCWQYNPKMR
INSR     223  TSLA.EQPYQGLS.NEQVLKFVMDGGY.......LDQPDNCPERVTDLMRMCWQFNPKMR
IRR      191  VTLA.EQPYQGLS.NEQVLKFVMDGGV.......LEELEGCPLQLQELMSRCWQPNPRLR
KDR      236  FSLG.ASPYPGVKIDEEFCRRLKEGTR.......MRAPDYTTPEMYQTMLDCWHGEPSQR
KIT      264  FSLG.SSPYPGMPVDSKFYKMIKEGFR.......MLSPEHAPAEMYDIMKTCWDADPLKR
LTK      195  FSLG.YMPYPGRT.NQEVLDFVVGGGR.......MDPPRGCPGPVYRIMTQCWQHEPELR
MERTK    202  ATRG.MTPYPGVQ.NHEMYDYLLHGHR.......LKQPEDCLDELYEIMYSCWRTDPLDR
MET      232  MTRG.APPYPDVN.TFDITVYLLQGRR.......LLQPEYCPDPLYEVMLKCWHPKAEMR
MST1R    222  LTRG.APPYRHID.PFDLTHFLAQGRR.......LPQPEYCPDSLYQVMQQCWEADPAVR
NTRK1    225  FTYG.KQPWYQLS.NTEAIDCITQGRE.......LERPRACPPEVYAIMRGCWQREPQQR
NTRK2    219  FTYG.KQPWYQLS.NNEVIECITQGRV.......LQRPRTCPQEVYELMLGCWQREPHMR
NTRK3    206  FTYG.KQPWFQLS.NTEVIECITQGRV.......LERPRVCPKEVYDVMLGCWQREPQQR
PDGFRA   261  FSLG.GTPYPGMMVDSTFYNKIKSGYR.......MAKPDHATSEVYEIMVKCWNSEPEKR
RET      217  VTLG.GNPYPGIP.PERLFNLLKTGHR.......MERPDNCSEEMYRLMLQCWKQEPDKR
ROR1     205  FSFG.LQPYYGFS.NQEVIEMVRKRQL.......LPCSEDCPPRMYSLMTECWNEIPSRR
ROR2     201  FSYG.LQPYCGYS.NQDVVEMIRNRQV.......LPCPDDCPAWVYALMIECWNEFPSRR
RYK      223  MTLG.QTPYVDID.PFEMAAYLKDGYR.......IAQPINCPDELFAVMACCWALDPEER
TEK      211  VSLG.GTPYCGMT.CAELYEKLPQGYR.......LEKPLNCDDEVYDLMRQCWREKPYER
TIE1     198  VSLG.GTPYCGMT.CAELYEKLPQGYR.......MEQPRNCDDEVYELMRQCWRDRPYER
TYRO3    196  MTRG.QTPYAGIE.NAEIYNYLIGGNR.......LKQPPECMEDVYDLMYQCWSADPKQR
                 305  310  315  320  325  330  335  340  345  350  355  360
```

```
                       α8              η6
EGFR                   llllllllllll    lll
EGFR     264  PKFRELIIEFSKMARDPQRYLVIQGDDEEDMDDVVDADEYLIPQ
ALK      262  PNFAIILERIEYCTQDPDVINTALPIEY................
AXL      244  PSFTELREDL..................................
CSF1R    291  PTFQQICSFLQEQA..............................
DDR1     291  PPFSQLHRFLAEDALNT...........................
DDR2     265  PSFQEIHLLL..................................
EPHA1    232  PHFQK.......................................
EPHA10   229  PRFSQIHS....................................
EPHA2    273  PKFADIVSILDKLI..............................
EPHA3    253  PKFEQIVSILDKLIRNPGSLKIITSNLLLD..............
EPHA4    235  PKFGQIVN....................................
EPHA5    260  PKFDEIVNMLDKLIRNPAHHHHHH....................
EPHA7    256  PKFEQIVGILDKMIRNPNSAHH......................
EPHA8    235  PRFSQIVS....................................
EPHB1    250  PRFAEIVNTLDKMIRNPASLKTVA....................
EPHB2    226  PKFGQIVNTLDKMIRNPNSLKAMA....................
EPHB3    252  PKFSQIVNTLDKLIRNPASLKVI.....................
EPHB4    250  PRFPQIVSALDKMIRNPASLKIVAR...................
EPHB6    231  PHFDQ.......................................
ERBB2    260  PRFRELVSEFSRMARDPQRFVVIQNEPLDSTFYRSLL.......
ERBB3    235  PTFKELANEF..................................
ERBB4    258  PKFKELAAEFSRMARDPQRYLVIQGDDRM...............
FGFR1    275  PTFKQLVEDLDRIVALTSNQE.......................
FGFR2    271  PTFKQLVEDLDRILTLT...........................
FGFR3    277  PTFKQLVEDLDRVLTVTS..........................
FGFR4    259  PTFKQLVEALDKVLLAV...........................
FLT1     275  PRFAELVEKLGDLL..............................
FLT3     297  PSFPNLTSFLGCQL..............................
IGF1R    273  PSFLEIISSIKEEMEPGFREVSFYYSEENK..............
INSR     274  PTFLEIVNLLKDDLHPSFPEVSFFHSEENK..............
IRR      242  PSFTHILDSIQE................................
KDR      288  PTFSELVEHLGNLLQANA..........................
KIT      316  PTFKQIVQLIEKQISES...........................
LTK      246  PSFASILERLQY................................
MERTK    253  PTFSVLRLQLERLLESLP..........................
MET      283  PSFSELVSRISAIFSTFIG.........................
MST1R    273  PTFRVLVGEVEQIVSALLGDHYVQLP..................
NTRK1    276  HSIKDVHARLQALAQAPPVYLDVL....................
NTRK2    270  KNIKGIHTLLQNLAKASPVYLDILG...................
NTRK3    257  LNIKEIYKILHALGKATPIY........................
PDGFRA   313  PSFYHLSEIVENLLPGQYKKSYEKIHLDFLKSD...........
RET      268  PVFADISKDLEKMMVKR...........................
ROR1     256  PRFKDIHVRLRSW...............................
ROR2     252  PRFKDIHSRLRAWG..............................
RYK      274  PKFQQLVQCLTEFHAALGAYV.......................
TEK      262  PSFAQILVSLNRMLEERKTYVNTTLYEKFTYAGIDCSAE.....
TIE1     249  PPFAQIAL....................................
TYRO3    247  PSF.........................................
                 365  370  375  380  385  390  395  400
```
